# When Cell-Mediated Immunity after Vaccination Is Important

**DOI:** 10.3390/pathogens13010065

**Published:** 2024-01-09

**Authors:** Roberto Paganelli

**Affiliations:** 1 Internal Medicine, UniCamillus, International Medical University in Rome, 00131 Rome, Italy; roberto.paganelli@unicamillus.org; 2 YDA, Institute of Clinical Immunotherapy and Advanced Biological Treatments, 66100 Pescara, Italy

The review by Reeg D.B. and colleagues from the Freiburg University Medical Center was published in early January 2023 [1], addressing the often forgotten important issue of specific cellular antiviral responses provided by T lymphocytes in immunocompromised individuals. This is a general problem with all viral infections, as well as with antiviral vaccinations, and it is largely due to the unavailability of reliable and convenient specific assays. In fact, most studies rely on Elispot tests measuring interferon-γ-secreting T cells, which are not provided in most laboratories, as is the case for specific MHC-tetramer techniques using different epitopes. Therefore, unlike serological responses, for which handy and affordable kits are universally available, very few data exist on a large scale. It is generally known that some conditions such as cancer (solid or hematological), HIV infection and solid organ transplantation (SOT), requiring robust immunosuppression, induce a functional state of cellular immunodeficiency which impairs T cell responses to foreign antigens. However, these conditions vary for the type and degree of compromised functions, and each antigen requires a separate assessment, depending on the contribution of T cell subsets to the final response. The authors seek to analyze data from studies on T cell immune responses to SARS-CoV-2 (either through infection or vaccination) in three groups of immunocompromised patients up to the third quarter of 2022. The very initial data in the course of the pandemic seemed to imply significantly impaired SARS-CoV-2-specific immune responses after both natural infection and vaccination. Hence, high-risk groups deserve particular consideration for this reason and also to guide clinical decisions and therapeutic strategies.

In sarbecovirus infections, as in most viral diseases, several studies have shown that T cells are activated early and are associated with effective viral elimination, as well as affording protection against variants arising during the pandemic. This has been confirmed later in many studies on the landscape of cell-mediated responses to SARS-CoV-2 [2,3,4,5].

Many studies analyzed by the authors have subsequently confirmed this, despite the fact that more variants, rounds of vaccinations with different subtypes and platforms, and therapeutic measures have been adopted in the three years elapsed since the beginning of the pandemic.

The significant role of T cell responses has also been validated in the case of exposure to viral variants of SARS-CoV-2 due to largely conserved epitopes across different variants and the breadth of epitopes recognized by T cells in every individual [6].

## 1. Boosting T Responses

The boosted response considered by the authors has not yet been complicated by the large heterogeneity in the immune imprinting of different individuals, which has been delineated in subsequent studies [7], showing the importance of temporal sequences of viral variant infection and vaccination for long-lasting cross-reactive protection. A previous study on booster vaccine doses in nursing home residents in Italy has shown similar results, with boosted restoration of spike-specific T cell responses in SARS-CoV-2 naive residents who responded poorly to the first immunization, while those with a previous infection had a major increase in the magnitude of vaccine-induced cell-mediated immunity, and at earlier time points [8]. The group in Freiburg, together with others, has already reported that booster vaccination increased the breadth of the spike-specific T cell response in convalescent individuals but not in vaccinees with complete initial vaccination; however, in both, the targeted T cell epitopes were broadly conserved [9]. Rapid reactivation of CD8+ T cell responses after boosters was also detected by Reinscheid et al. [10], with long-term preservation of spike-specific T cell immunity also targeting emerging variants. In liver transplant receivers under immunosuppressive therapy, this booster effect was somewhat blunted [11], with a reduced frequency and epitope repertoire compared to healthy controls, in part due to a decreased frequency of spike-reactive follicular T helper cells. A similar observation applied to patients with multiple sclerosis, where the T cell response exhibited a significant increase after boosting, without a significant change in the number of responders [12]. However, 93% of fingolimod-treated patients only exhibited a humoral-specific response.

## 2. Response in Cancer

Variations in the immune profiles of cancer patients after vaccination or natural infection have been described, along with the differences among those with solid tumors and those with hematologic malignancies [1]. In a prospective study of a comprehensive immune profiling of cancer patients without intensive chemo/immunotherapy, Tembhare et al. [13] showed severely depleted circulating myeloid DCs and helper T subsets in the initial phase of infection which were strongly associated with severe COVID-19 independent of age, type of comorbidity, and other parameters. Notably very few studies have addressed the point in question in 2023, with the exception of Saavedra et al. [14], who showed a blunted CD4 T cell response after vaccination of breast cancer female patients receiving cdk inhibitors who experienced breakthrough infections. Therefore, the paper by Reeg et al. [1] represents a solid unique reference for the assessment of this high-risk group.

## 3. Response in HIV

Most of the findings reported in this field were not further studied since Reeg and coworkers’ publication [1], which indicated lower frequencies of SARS-CoV-2-specific CD4+ T cells in patients with unsuppressed HIV infections compared to those with controlled viremia, but no differences for the CD8+ T cell levels, and that booster vaccination was able to curb the negative impact of low CD4+ T cell counts, which also impacted the humoral response. A prospective cohort study of T cell responses after two vaccine doses in people living with HIV has found lowered CD4 and CD8 responses, with breakthrough infections (only one requiring hospitalization) in those with lower responses [15]. This is further but still insufficient evidence for a higher risk in this group of patients, and therefore more investigations are required.

## 4. Response in SOT

The part of the review by Reeg et al. [1] referring to the response in SOT had several important practical consequences, since many subtle impairments of the immune response in SOT could be identified. Among them were faster decreasing frequencies of virus-specific T cells and lower virus-specific cellular responses compared to other risk groups, such as patients with primary immunodeficiencies or HIV infection, which suggests the possible temporary modification or suspension of the treatment that aims to improve the vaccine-elicited adaptive immune response in this endangered population. This point was also raised by Cremoni et al. [16], who showed that low T cell responsiveness at baseline was a major independent risk factor for progression to severe pneumonia in kidney transplant recipients in a multicentric prospective study. However, LaCivita and coworkers [17] reported comparable levels of adaptive immune responses to three doses of vaccine in healthy donors and in two cohorts of fragile patients (common variable immunodeficiency and kidney transplant recipients). In a subsequent study investigating T cell responses of SOT recipients, lower levels were found in those without prior SARS-CoV-2 infection, but the levels were comparable to controls in SOT recipients with previous infection [18]. Longer follow-up studies and updated vaccination schedules are needed to validate these findings and explore the possibility of changing immunosuppressive therapeutic regimens in this high-risk group.

## 5. Corroborating Data from Autoimmune Immunosuppressed and Primary Immunodeficient Patients

The importance of T cell responses after vaccination has been gaining attention throughout the year since the publication of the paper by Reeg et al. [1], mainly in studies of the profile of the functional spectrum of hybrid T cell immunity. With the use of novel technologies, Cai et al. [19] have confirmed the essential role played by T cells in providing enhanced protection against subsequent episodes of COVID-19. Therefore, studies analyzing only antibody responses may miss an essential component of the correlate of protection afforded by vaccination, as well as misinterpret the risk in immunocompromised patients. New corroborating evidence for this was found in studies of vaccination in patients affected by primary immunodeficiencies, where Amodio and coworkers [20] had already indicated that a strong cellular response could be observed in these patients and therefore studies of T cell immunity should complement serology to assess the real risk. Recently, Paris [21] seems to have overlooked this point in his article summarizing vaccine responses in primary immunodeficient patients, thus labelling this group at higher risk despite cellular responses after vaccination in many of them (e.g., 67% to 83% of those with a common variable immunodeficiency), which is the basis for the recommendation for influenza vaccination of this group of patients.

Another group of immunocompromised patients are those affected by autoimmune diseases requiring immunosuppressive therapies. Even in these cases, steady T cell responses to SARS-CoV-2 vaccination have been found at levels similar to controls [22], and were found to be present after a booster dose, therefore appearing to be protective against subsequent reinfections.

## 6. Conclusions

As stated by Reeg et al. [1], “current data imply booster vaccinations as important measures leading to an enhanced SARS-CoV-2-specific immunity in immunocompromised patients. While first results suggest a particularly improved humoral response, a positive effect on virus-specific T cells is likely, but less well-characterized and, therefore, requiring further analyses”. We should include an assessment of T cell responses when evaluating the correlates of protection of vaccination, more so in immunocompromised patients, those who are unable to produce antibody responses, and those who are undergoing B cell-directed therapies such as Rituximab [22]. Despite recent reviews from eminent vaccinologists also failing to acknowledge this [23], several cutting-edge studies have appeared in the second half of this year, pointing to the essential protection afforded by boosting T cell immunity, which seems to be stimulated to a greater extent when vaccination is given to previously infected subjects [19,24]. However, no reference standards nor validated methods to measure protective levels of cellular immunity are available, so this remains an urgent knowledge gap to be filled. In the meantime, more studies in these patient groups with impaired immune responses should be encouraged, since they may also shed light on the roles of CD4 and CD8 T cell-mediated immunity in response to vaccinations and booster doses in other contexts.
